# Fasting impairs type 2 helper T cell infiltration in the lung of an eosinophilic asthma mouse model

**DOI:** 10.1002/2211-5463.13268

**Published:** 2021-08-20

**Authors:** Yusuke Suzuki, Tomoya Hayashi, Ryoma Yokoyama, Fumika Nakagawa, Joe Inoue, Taishi Higashi, Risako Onodera, Keiichi Motoyama

**Affiliations:** ^1^ Faculty of Pharmacy Graduate School of Pharmaceutical Sciences Kumamoto University Kumamoto Japan; ^2^ Division of Vaccine Science Department of Microbiology and Immunology The Institute of Medical Science The University of Tokyo (IMSUT) Tokyo Japan; ^3^ International Research and Development of Microbiology and Immunology IMSUT Tokyo Japan; ^4^ Laboratory of Mock Up Vaccine Center for Vaccine and Adjuvant Research (CVAR) National Institutes of Biomedical Innovation, Health and Nutrition (NIBIOHN) Osaka Japan; ^5^ Faculty of Pharmacy Graduate School of Pharmaceutical Science Keio University Tokyo Japan; ^6^ Institute for Advanced Biosciences Keio University Tsuruoka Japan; ^7^ Systems Biology Program Graduate School of Media and Governance Keio University Fujisawa Japan; ^8^ Priority Organization for Innovation and Excellence Kumamoto University Kumamoto Japan

**Keywords:** eosinophils, eosinophilic asthma, fasting, IL‐33, lung infiltration, Th2

## Abstract

Eosinophilic asthma is a form of bronchial asthma that is caused by the pulmonary infiltration of eosinophils and accounts for approximately half of the patients with severe asthma. Several cell types of the immune system in synergy with the epithelial cells of the lung provoke an inflammatory response in patients with asthma. Recently, the effect of fasting on immune cells and inflammation has attracted considerable attention. Therefore, we examined whether fasting may serve as novel preventive strategy in patients with asthma. In our study, we employed a previously established mouse model of eosinophilic asthma. C57BL/6 mice were inoculated intranasally with interleukin‐33 and ovalbumin (OVA) in order to induce eosinophil infiltration in the lung and subjected to a 48‐h long fasting period directly after or 7 days postinoculation. We used flow cytometry to characterise infiltrated immune cells in the lung and measured the quantity of inflammatory cytokines as well as antigen‐specific immunoglobins (Ig) by ELISA. Our results indicated that fasting lowered the number of eosinophilic pulmonary infiltrates in the eosinophilic asthma model mice. Furthermore, fasting suppressed anti‐OVA IgG1 production. Fasting suppressed Th2 cytokine production by impairing Th2 accumulation in the lung. The findings suggest that fasting may be a novel preventive strategy for eosinophilic asthma.

## Graphical abstract

The pulmonary infiltration of eosinophils exacerbates asthma symptoms. Here, we found that fasting suppresses eosinophil infiltration in eosinophilic asthma model mice induced by OVA and IL‐33. Furthermore, the number of Th2 cytokine‐producing cells, which play a critical role in eosinophil infiltration, was reduced by fasting. These findings suggest fasting has potential as a novel prophylactic treatment for eosinophilic asthma.

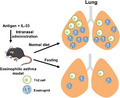

**Abbreviations**
AHR: airway hyper‐reactivity
AMPK: AMP‐activated protein kinase
BALF: bronchoalveolar lavage fluid
BM: bone marrow
Glut1: glucose transporter 1
IG: immunoglobins
IL: interleukin
ILC2: type 2 innate lymphoid cells
mTORC: mammalian target of rapamycin complex
mLN: mediastinal lymph node
TSLP: thymic interstitial lymphopoietin
Th2: type 2 helper T
OVA: Ovalbumin

Asthma is characterised by airway inflammation, airway hyper‐reactivity (AHR), mucus overproduction and chronic eosinophilic inflammation [1], and it affects over 300 million people worldwide [2]. Several cells of the innate and adaptive immune system work together with epithelial cells to provoke an inflammatory response [3]. Aeroallergens, such as house dust mites and pollen, damage the type 2 epithelial cells of the lung, resulting in the release of alarmin molecules such as interleukin‐33 (IL‐33), thymic interstitial lymphopoietin (TSLP) and IL‐25. These alarmins activate the immune cells and induce an inflammatory response [4]. IL‐33 plays critical roles in both innate and adaptive immune responses in the mucosal organs [5]. IL‐33 induces the production of cytokines such as IL‐4, IL‐5 and IL‐13 by activating type 2 innate lymphoid cells (ILC2), mainly in the innate immune system [6], and type 2 helper T (Th2) cells in the adaptive immune system [7]. IL‐4 is important for the production of IgE from B cells [8] which in turn induces an allergic reaction. IL‐5 induces eosinophil differentiation and migration [9]. IL‐13 induces AHR and mucus hypersecretion [10]. As these immune responses induce the symptoms of asthma, regulation of the immune response is the primary strategy in asthma treatment.

Eosinophils, also known as granulocytes, are inflammatory cells that produce basic proteins, reactive oxygen species and lipid mediators. The granular proteins that are released in the lung tissue cause damage to the epithelial and mucosal cells, resulting in the induction of AHR and mucus hypersecretion. Eosinophils not only provoke a transient inflammatory response but also induce irreversible airway narrowing. Airway narrowing occurs because eosinophils release various growth factors and fibrosis mediators, which promote the thickening of the airway wall (airway remodelling) as a result of airway tissue destruction and degeneration [11, 12]. Furthermore, a positive correlation between eosinophilic airway inflammation and the severity of asthma has also been reported [13]; eosinophils are considered to be involved in chronic and severe asthma pathology. Thus, the suppression of eosinophil infiltration into the lung tissue is critical in improving the pathological symptoms of eosinophilic asthma.

Although a majority of patients with asthma can be treated using a combination of inhaled corticosteroids (ICS) and a short‐ or long‐acting β_2_‐adrenergic agonist (LABA), approximately 5% of patients with severe asthma show inadequate response to their treatment regimen [14]. Currently, patients with severe asthma are prescribed biologics depending on the phenotype that is identified using certain biomarkers (sputum and blood eosinophil counts, serum IgE, exhaled nitric oxide, and serum periostin) [15]. For example, omalizumab is an anti‐IgE antibody [16] and dupilumab is an antibody that is generated against the α chain of the IL‐4 receptor [17]. In addition, mepolizumab is an anti‐IL‐5 antibody, and benralizumab is an antibody generated against the IL‐5 receptor, which induces a decrease in eosinophil concentration [18, 19]. However, the use of these biologics has been limited as they are expensive [20]. Furthermore, asthma is a heterogeneous disease with multiple phenotypes that limits the use of personalised medicine in terms of biologics [15].

Fasting is a form of dietary intervention that involves a halt in the consumption of food for a certain period of time. Upon > 24 h of fasting, humans, rodents and other mammals begin using reserve fat as an energy source instead of glucose. Free fatty acids disintegrate from the adipose tissue and are converted into ketone bodies that are used as an energy source. Findings from controlled investigations in experimental animals, as well as emerging human studies, indicate that fasting may provide effective strategies to reduce weight, delay ageing and optimise health. In addition, fasting has been reported to display a protective effect against a variety of diseases such as diabetes, cancer, heart disease and neurodegenerative diseases [21]. Therefore, the effect of fasting on immune cells has attracted attention of several researchers in recent years. For example, fasting has been reported to ameliorate the symptoms of multiple sclerosis by suppressing the production of self‐reactive T cells and increasing regulatory T cell production [22]. Fasting also reduces T cells in the spleen and increases in the bone marrow [23].

Thus, changes in nutritional status brought about by fasting might regulate host immune responses and prevent the symptoms of asthma. In fact, calorie restriction reportedly lowers the concentration of inflammatory markers and oxidative stress in asthmatics [24], and it also reduces allergic reactions to house dust mites in rats [25]. Regulation of immune cells through nutritional intervention is mediated through various mechanisms such as cell death due to energy shortage [26] and regulation by gut microbiota [27]. Therefore, there exists clear evidence that the nutritional status of the host affects the immune system and that dietary interventions may alleviate asthma symptoms. However, the mechanisms by which nutritional intervention through fasting affects the Th2 cells and eosinophils involved in eosinophilic asthma pathology remain unclear.

In this study, we analysed the effects of fasting on immune cells, especially eosinophils, in the lungs. Eosinophil infiltration was induced into the lungs of mice immunised with ovalbumin (OVA) and IL‐33, as model antigen and adjuvant, respectively; however, they were significantly suppressed upon fasting. Moreover, fasting did not affect the induction of Th2 cells that typically induce the lung infiltration of eosinophils, but decreased the Th2 cell activation in the lungs.

## Materials and methods

### Pernasality immunisation with OVA and IL‐33

Eosinophilic asthma model was created by intranasally inoculating six‐week‐old C57BL/6 mice (female) with 20 μL OVA (0.5 mg·mL^−1^) and recombinant mouse IL‐33 protein, carrier free (5 μg·mL^−1^) (R&D systems, MN, USA) in PBS on 0 and 7 day. Mice were fasted for 48 h from day 0 to day 2 or from day 7 to day 9. Bronchoalveolar lavage fluid (BALF), serum, mediastinal lymph node (mLN), lung, spleen and bone marrow (BM) samples were collected on day 10. All animal experiments in this manuscript were carried out in accordance with both the ARRIVE Guideline for reporting *in vivo* experiments and the guidelines approved by the Ethics Committee for Animal Care and Use of Kumamoto University composed of a third party (Approval ID: A020‐030). All experimental animals were bred in a 12‐h light‐dark cycle environment with free access to food and water except during the experimental period. To minimise the pain or suffering, the animal experiments were performed under appropriate anaesthesia and analgesia to minimise pain.

### Preparation of BAL, mLN, lung, spleen and BM cells

The lung was washed twice with 1.2 mL PBS, and the BAL cells were collected. The lungs were then minced and stirred in 3 mL RPMI 1640 medium (Nacalai Tesque, Kyoto, Japan) containing 100 U·mL^−1^ penicillin, 100 μg·mL^−1^ streptomycin and 2‐mercaptoethanol supplemented with 10% FBS, 200 U·mL^−1^ Collagenase (Fujifilm, Tokyo, Japan) and 100 U·mL^−1^ DNase I (Fujifilm) for 1 h at 37 °C. The mLN, lung and spleen cells were obtained by grinding tissues. The femurs of mice were collected, both ends were amputated and then washed with RPMI 1640 medium with 10% FBS to collected BM cells. The mLN, lung, spleen and BM cells were filtrated through 40 μm cell strainer. Red blood cells were removed by ACK lysis buffer, and then, cells were suspended in RPMI medium with 10% FBS. These cells were counted using LUNA II automated cell counter (Logos Biosystems, South Korea).

### Flow cytometry

For surface staining, nonspecific binding was blocked with anti‐CD16/CD32 antibody (clone: 93; BioLegend, CA, USA). Zombie Aqua Fixable Viability Kit (BioLegend) was also added to discriminate dead cells. After that, it was stained with fluorochrome‐conjugated antibodies; Siglec‐F (clone: E50‐2440; BD Biosciences, Franklin Lakes, NJ), CD11b (clone: M1/70; BioLegend), CD8a (clone: 53‐6.7; BioLegend), CD4 (clone: RM4‐5; BioLegend), Ly6G (clone: 1A8; BioLegend), CD11c (clone: N418; BioLegend), CD19 (clone: 6D5; BioLegend), CD45 (clone: 30‐F11; BioLegend), Lineage (145‐2C11, RB6‐8C5, RA3‐6B2, Ter‐119, M1/70, BioLegend), Thy‐1.2 (clone: 30‐H12; BioLegend), Sca‐1 (clone: D7; BioLegend), ST2 (clone: DIH4; BioLegend), CD44 (clone: 1M7; BioLegend) and CD62L (clone: MEL‐14; BioLegend). The stained samples were analysed using Aria IIu flow cytometers with DIVA software (BD Biosciences) and FlowJo software version 10 (FlowJo LLC, Ashland, OR).

### Cell culture

The mLN cells (1 × 10^5^ cells/wells) were cultured in RPMI 1640 medium (Nacalai Tesque) containing 100 U·mL^−1^ penicillin, 100 μg·mL^−1^ streptomycin and 2‐mercaptoethanol supplemented with 10% FBS at 37 °C in humidified 5% CO_2_. Cells were stimulated with 100 μg·mL^−1^ OVA for 5 days. The supernatant was collected for the measurement of cytokine production from mLN cells.

### Detection of cytokine productions by ELISA

The amount of IL‐5, IL‐13 or IFN‐γ in BALF or culture medium was determined according to the protocol of by ELISA MAX^TM^ standard set (Biolegend) or DuoSet ELISA kit (R&D systems) with slight modification. ELISA 96‐well half plates (Corning, NY, USA) were coated with anticytokine antibody (IFN‐γ (BioLegend), IL‐5 (BioLegend) and IL‐13 (R&D systems)) in 0.1 N carbonate buffer at 4 °C overnight. After washing three times with PBS containing 0.05% Tween 20 (PBS‐T), the wells were blocked with 1% BSA in PBS for 1 h at room temperature. Samples and standards were added. Nonstimulated samples with OVA were included as a negative control. After incubation at room temperature for 2 h, the wells were washed three times with PBS‐T. Biotin‐conjugated anti‐cytokine antibody was used as detection antibody. After incubation at room temperature for 1 h, the plates were extensively washed with PBS‐T. Streptavidin–horseradish peroxidase (HRP) was added. After washing three times with PBS‐T, specific cytokine binding was visualised by adding TMB solutions, and then, the reaction was terminated by 1 N H_2_SO_4_. Finally, the absorbance at 450 nm was measured by microplate reader (Epoch, BioTeK, CA, USA).

### Quantification of antigen‐specific IgG1 by ELISA

ELISA 96‐well half plates were coated with OVA (10 μg·mL^−1^) in 0.1 N carbonate buffer at 4 °C overnight. After washing three times with PBS containing 0.05% Tween 20 (PBS‐T), the wells were blocked with 1% BSA in PBS for 1 h at room temperature. Serum samples were serially diluted with blocking buffer and added to the plates. After incubation at room temperature for 2 h, the wells were washed three times with PBS‐T. Then, HRP‐conjugated anti‐mouse IgG1 antibody (Southern Biotech, AL, USA) was added. After incubation at room temperature for 1 h, the plates were extensively washed with PBS‐T. TMB solution was added, and then, the reaction was terminated by 1 N H_2_SO_4_. Finally, the ELISA plates were measured by microplate reader (Epoch, BioTeK, CA, USA). Titres of antigen‐specific IgG1 was determined by log‐linear interpolation of the serum dilution value corresponding to cut‐off absorbance (OD450 of 0.2).

### Quantification of antigen‐specific IgE by ELISA

ELISA 96‐well half plates were coated with purified antimouse IgE (0.5 mg·mL^−1^) in PBS at 4 °C overnight. After washing three times with PBS containing PBS‐T, the wells were blocked with 1 x Block Ace (KAC, Kyoto, Japan) in PBS for 1 h at room temperature. Serum samples and anti‐OVA IgE (clone: 2C6; Bio‐Rad) as a standard were added. After incubation at room temperature for 2 h, the wells were washed three times with PBS‐T. Then, 10 μg·mL^−1^ of HRP‐conjugated OVA (Bio‐Rad, CA, USA) diluted with 1 × Block Ace was added. After incubation at room temperature for 1 h, the plates were extensively washed with PBS‐T. TMB solution was added, and then, the reaction was terminated by 1 N H_2_SO_4_. Finally, the ELISA plates were measured by microplate reader (Epoch, BioTeK, CA, USA).

### Statistical analyses

Data are supplied as the mean ± S.E.M. Statistical significance was determined by a Scheffe’s test after performing an ANOVA test using StatView (SAS Institute, Inc., NC, USA). *P < *0.05 was considered statistically significant.

## Results

### Effect of fasting on the pulmonary infiltration of eosinophils

**Fig. 1:**
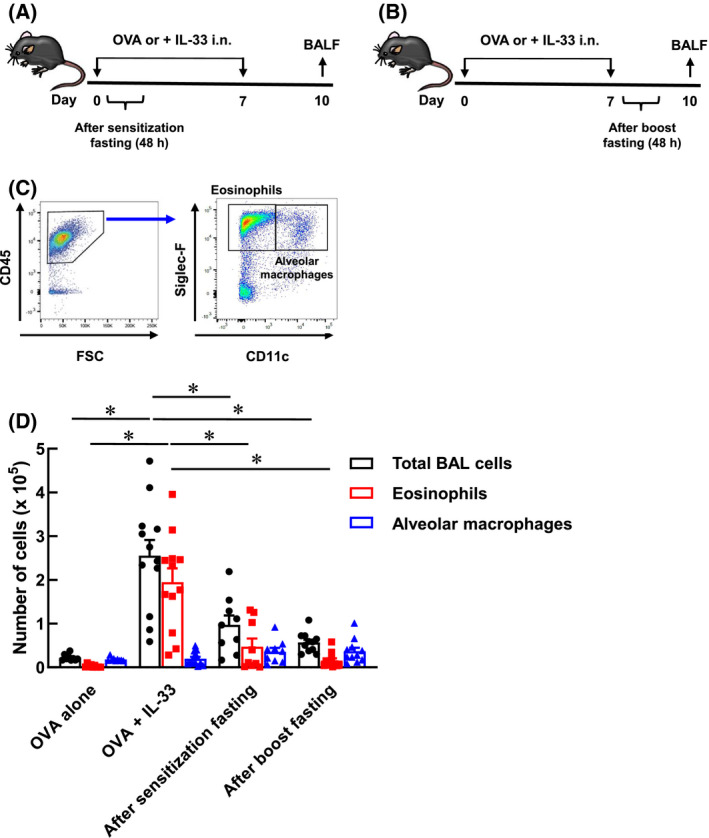
Suppression of pulmonary infiltration of eosinophils by fasting. (A) Six‐week‐old C57BL/6 mice were inoculated 20 μL of PBS solution containing OVA (0.5 mg·mL^−1^) and IL‐33 (5 μg·mL^−1^) into noses on days 0 and 7. The mice were fasted for 48 h from day 0 to day 2. (B) The mice were fasted for 48 h from day 7 to day 9. (C) Gating strategy after the exclusion of doublets, dead fluorescent‐positive cells were shown. CD45^+^ Siglec‐F^+^ CD11c^−^ cells (eosinophils) and CD45^+^ Siglec‐F^+^ CD11c^+^ cells (alveolar macrophages) were analysed by flow cytometry. (D) Each column represents the total BAL cells (black), eosinophils (red) and alveolar macrophages (blue). Each value represents the mean ± S.E.M. of 9‐12 experiments. *P < 0.05 compared with each group. Statistical significance was determined by Scheffe’s test after performing an ANOVA test using StatView (SAS Institute, Inc., NC, USA).

First, to examine whether fasting is effective in preventing the symptoms of eosinophilic asthma, we evaluated the number of eosinophils in the bronchoalveolar lavage fluid (BALF) of model mice with eosinophilic asthma after fasting. Mouse models of eosinophilic asthma were established by the intranasal administration of a solution containing 10 μg of OVA (as a model antigen) and 100 ng of IL‐33 (as an adjuvant) to C57BL/6 mice on days 0 and 7. We confirmed that intranasal administration of OVA alone did not induce pulmonary infiltration of eosinophils (data not shown). At this time, the mice were subjected to fasting for 48 h at either day 0 (after sensitisation fasting: Fig. 1A) or 7 (after boost fasting: Fig. 1B). BALF was collected on day 10, and eosinophils and alveolar macrophages in the BALF were measured using flow cytometry. After the exclusion of dead cells, the CD45^+^ Siglec‐F^+^ CD11c^‐^ cells and CD45^+^ Siglec‐F^+^ CD11c^+^ cells were measured as eosinophils and alveolar macrophages, respectively (Fig. 1C). As a result, the OVA + IL‐33 mice that were not subjected to fasting did not show any alteration in the number of alveolar macrophages compared to that in OVA‐alone mice; however, the number of eosinophils increased significantly, suggesting the pulmonary infiltration of eosinophils. In addition, the increase in eosinophils in the OVA + IL‐33 mice was significantly suppressed as a result of fasting after both the first and booster stimulation (Fig. 1D). These results suggest that fasting may affect both innate and adaptive immunity as an innate immune response was observed after nasal administration on day 0, whereas an adaptive immune response was observed after nasal administration on day 7. Collectively, fasting acts on both innate and adaptive immunity, and it may have preventive effects on eosinophilic asthma by suppressing the pulmonary infiltration of eosinophils. In subsequent studies, to examine the effect of fasting on adaptive immunity, the mice were subjected to after boost fasting.

### Effect of fasting on IgG1 and IgE production

**Fig. 2:**
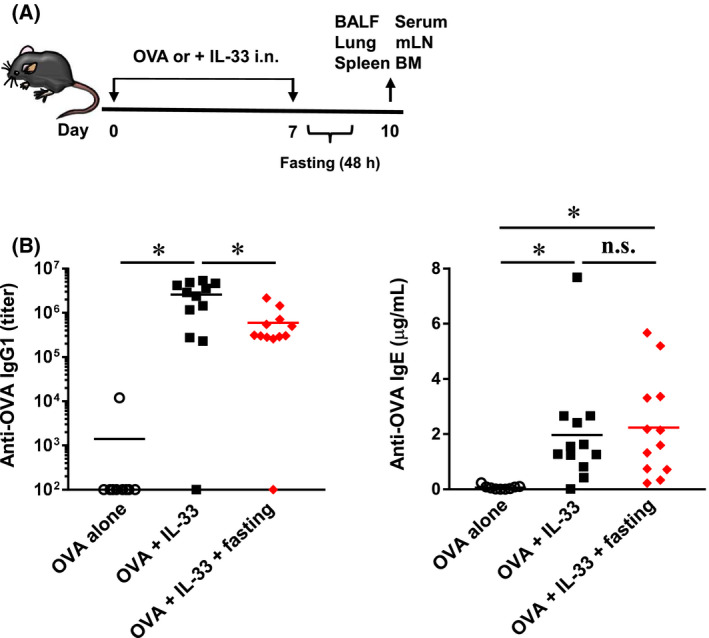
Effect of fasting on anti‐OVA total IgG1 and IgE production. (A) Six‐week‐old C57BL/6 mice were inoculated 20 μL of PBS solution containing OVA (0.5 mg·mL^−1^) and IL‐33 (5 μg·mL^−1^) into noses on days 0 and 7. The mice were fasted for 48 h from day 7 to day 9. And then, the mice were refed for 24 h from day 9 to day 10. BALF, serum, mLN and lung were collected on day 10. (B) Anti‐OVA total IgG1 and IgE in the serum were determined by ELISA. Each bar represents the mean of 9‐12 experiments. *P < 0.05 compared with each group. Statistical significance was determined by Scheffe’s test after performing an ANOVA test using StatView (SAS Institute, Inc., NC, USA). n.s.; not significant.

Eosinophil and IgE levels have typically been used as biomarkers for asthma. IgG and IgE are produced by the plasma cells that are differentiated from B cells. In particular, IgE induces an allergic reaction by associating with the Fcɛ receptor on mast cells and cross‐linking with allergens. In the previous section, we established that fasting suppresses the number of eosinophils in BALF (Fig. 1). Therefore, we next investigated the effect of fasting on serum IgE levels and examined whether fasting is effective in eliciting not only cell‐mediated immunity but also humoral immunity. The mice were subjected to fasting for 48 h from day 7 to day 9 after the booster stimulation and refed from day 9 to day 10. Following this, the serum, BALF, lung, spleen, mediastinal lymph nodes (mLN) and bone marrow were collected on day 10 (Fig. 2A). Subsequently, the anti‐OVA IgG1 and anti‐OVA IgE levels in serum were measured using enzyme‐linked immunosorbent assay (ELISA). Results revealed an increase in the serum anti‐OVA IgG1 levels in OVA + IL‐33 mice; however, the levels were significantly suppressed in fasting mice (Fig. 2B). In contrast, an increase in the serum anti‐OVA IgE levels in OVA + IL‐33 mice was observed in fasting mice (Fig. 2B). These results suggest that IgE‐producing plasma cells might be less susceptible to fasting when compared to IgG1‐producing plasma cells.

### Production of Th2 cytokines after fasting

**Fig. 3:**
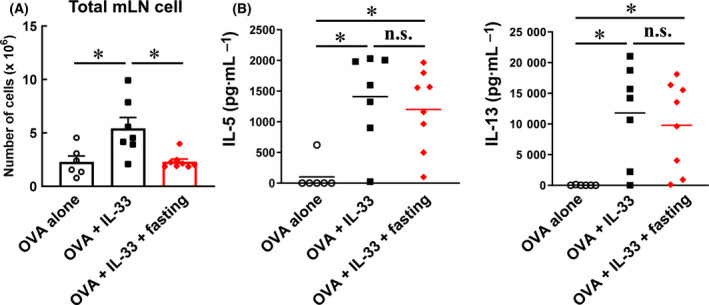
Effect of fasting on total mLN Cells and Their Ability of Th2 Cytokine Production. (A) The graph represents the number of total mLN cells. Each value represents the mean ± S.E.M. of 6–8 experiments. (B) The mLN cells (1 × 10^5^ cells/wells) were cultured in RPMI 1640 medium supplemented with 10% FBS. The mLN cells were stimulated with 100 μg·mL^−1^ OVA for 5 days. Then, IL‐5 and IL‐13 in culture supernatants were measured by ELISA. Each bar represents the mean of 6‐8 experiments. *P < 0.05 compared with each group. Statistical significance was determined by Scheffe’s test after performing an ANOVA test using StatView (SAS Institute Inc., NC, USA). n.s.; not significant.

Th2 cytokines play an important role in eliciting the pulmonary infiltration of eosinophils. Therefore, we hypothesised that fasting suppresses Th2 cytokine production in mice with asthma. Th2 cells differentiate and mature from naïve CD4^+^ T cells in the lymph nodes and gain the ability to produce Th2 cytokines. Therefore, we evaluated the effect of fasting on Th2 cell differentiation and maturation in these lymph nodes. First, we examined the effect of fasting on the number of immune cells in the mLNs. The mLNs were collected according to the protocol shown in Fig. 2A, and the number of mLN cells was counted. As shown in Fig. 3A, the number of mLN cells in OVA + IL‐33 mice was significantly higher compared to that in OVA‐alone mice. Moreover, the number of mLN cells in OVA + IL‐33 mice reduced significantly after fasting (Fig. 3A), suggesting that fasting might suppress the differentiation of naïve CD4^+^ T cells into Th2 cells.

Next, to investigate the effect of fasting on the maturation of differentiated Th2 cells, the amount of Th2 cytokines produced by Th2 cells was measured. The mLN cells collected were cultured under OVA stimulation for 5 days, and the IL‐5 and IL‐13 levels in the culture medium were quantified using ELISA (Fig. 3B). IL‐5 and IL‐13 levels showed a significant increase in Th2 cells derived from OVA + IL‐33 mice, suggesting the induction of OVA‐specific Th2 cells (Fig. 3B). However, IL‐5 and IL‐13 levels also increased in the Th2 cells derived from fasting mice. These results indicate that fasting does not affect the ability to produce Th2 cytokines from Th2 cells. Taken together, these results suggest that fasting might suppress the number of Th2 cells in mLN, but it does not affect cytokine production in differentiated Th2 cells.

### Fasting suppresses type 2 immunity in the lung

**Fig. 4:**
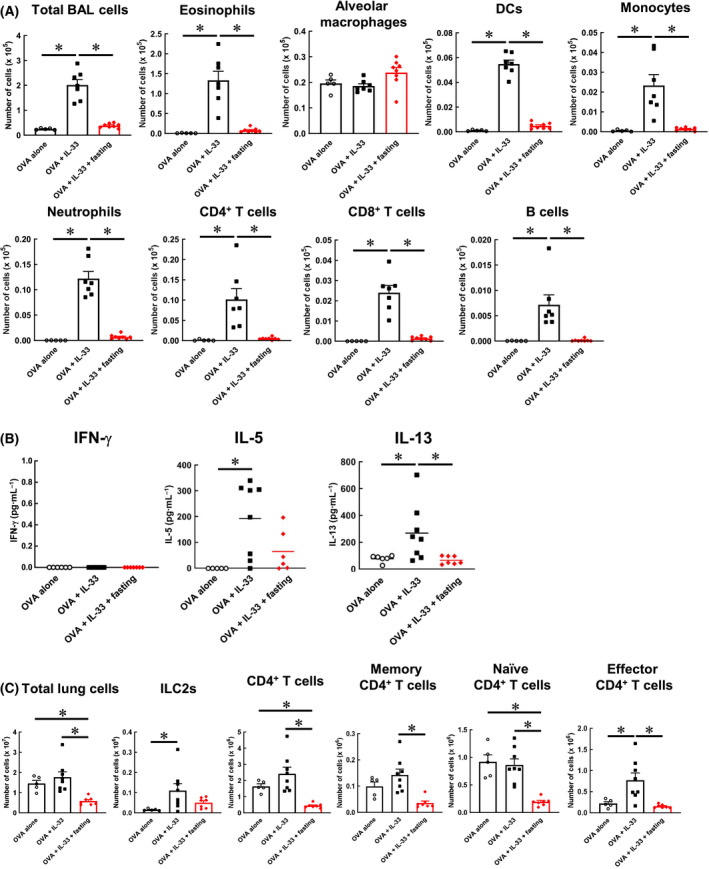
Fasting suppresses type 2 immunity in the lung. (A) The number of eosinophil, alveolar macrophage, DC, monocyte, neutrophil, CD4^+^ T cell, CD8^+^ T cell in BALF was determined by flow cytometry. (B) IL‐5, IL‐13 and IFN‐γ in BALF were measured by ELISA. Each bar represents the mean of 5–8 experiments. (C) The number of ILC2, CD4^+^ T cell, memory CD4^+^ T cell, naïve CD4^+^ T cell or effector CD4^+^ T cell in the lung was determined by flow cytometry. Each value represents the mean ± S.E.M. of 5–8 experiments. *P < 0.05 compared with each group. Statistical significance was determined by Scheffe’s test after performing an ANOVA test using StatView (SAS Institute, Inc., NC, USA). n.s.; not significant.

To gain insight into the mechanism of suppression of eosinophil infiltration into the lung brought about by fasting, the effect of fasting on the type 2 immune responses in the lung was investigated. Briefly, the BALF and lung tissues were collected as described in the protocol in Fig. 2A. Immune cells in BALF were measured using flow cytometry (Fig. S1). Fasting significantly suppressed not only the eosinophils but also other immune cells such as dendritic cells (DCs), monocytes, neutrophils, CD4^+^ T cells, CD8^+^ T cells and B cells (Fig. 4A). Next, to evaluate the effect of fasting on Th2 cytokines, Th1 cytokine (IFN‐γ) and Th2 cytokine (IL‐5 and IL‐13) levels in BALF were measured using ELISA. IFN‐γ levels were not significantly altered; however, the IL‐5 and IL‐13 levels were significantly suppressed as a result of fasting (Fig. 4B). Next, flow cytometry was used to measure the pulmonary infiltrates of ILC2 and effector CD4^+^ T cells, which produce Th2 cytokines and induce a type 2 immune response. After removing dead cells, the ILC2, CD4^+^ T cells, naïve CD4^+^ T cells and effector CD4^+^ T cells were identified as CD45^+^ ST2^+^ lineage^‐^ Thy‐1.2^+^ Sca‐1^+^ cells, CD45^+^ CD4^+^ cells, CD45^+^ CD4^+^ CD44^+^ CD62L^+^ cells, CD45^+^ CD4^+^ CD44^‐^ CD62L^+^ cells and CD45^+^ CD4^+^ CD44^+^ CD62L^‐^ cells, respectively (Fig. S2). As a result, the concentration of ILC2 in the lungs of fasting mice tended to be lower than that in OVA + IL‐33 mice (Fig. 4C). In addition, the increase in the number of lung effector CD4^+^ T cells in OVA + IL‐33 mice was significantly reduced as a result of fasting. These results suggest that fasting downregulates the type 2 immune response in the lung by suppressing the accumulation of Th2 cytokine‐producing cells such as ILC2 and effector CD4^+^ T cells.

### Effect of fasting on T cells in the spleen and bone marrow

Fasting suppresses eosinophil infiltration by reducing T cell accumulation in the lungs. However, it is unclear whether the reduction in T cell accumulation brought about by fasting is specific to the lung. Therefore, we evaluated the effects of fasting on T cells in tissues other than the lungs. The spleen is a lymphatic tissue rich in T cells. In the bone marrow, B cells are mainly differentiated and mature; however, T cells are also present in lower concentrations. Bone marrow is used for the temporary storage of T cells during fasting. First, the spleen and bone marrow were collected as described in the protocol in Fig. 2A. In each tissue, the CD45^+^ CD4^+^ cells, CD45^+^ CD4^+^ CD44^+^ CD62L^+^ cells, CD45^+^ CD4^+^ CD44^‐^ CD62L^+^ cells and CD45^+^ CD4^+^ CD44^+^ CD62L^‐^ cells were identified as CD4^+^ T cells, memory CD4^+^ T cells, naïve CD4^+^ T cells and effector CD4^+^ T cells, respectively. Results indicated that T cells in the spleen were suppressed because of fasting, similar to the observations in the lungs. However, there were no significant differences in the T cell concentration in the bone marrow between the OVA + IL‐33 mice and fasting mice. Collectively, our results indicated that although the inhibitory effect of fasting on immune cells was not lung‐specific, the immune response observed in the bone marrow was different from that in other tissues.

## Discussion

Our findings demonstrated that fasting might have prophylactic effect on eosinophilic asthma (Fig. 1D). Additionally, as fasting did not affect the production of IgE (Fig. 2B), the effect of fasting on allergic responses was relatively low. However, the pulmonary infiltration of eosinophils was completely suppressed, suggesting the potential of fasting as a new prophylactic alternative to biologics in combating eosinophilic asthma.

Eosinophils have been reported to exacerbate asthma symptoms by infiltrating the lungs and releasing various mediators [28]. In addition, eosinophils differentiate and mature in the bone marrow, express the IL‐5 receptor α subunit (IL‐5Rα) and circulate in the body. IL‐5, a chemokine, is recognised by IL‐5Rα and promotes eosinophil migration [29]. Therefore, IL‐5 may be a key target for suppressing the lung infiltration of eosinophils. Mepolizumab and benralizumab are antibody preparations that target IL‐5 and IL‐5Rα and have been used in the treatment of asthma [30, 31]. Therefore, we focused on the Th2 cytokine, IL‐5, as a key player in fasting induced suppression of eosinophil infiltration. Our results indicated that the amount of Th2 cytokines produced per Th2 cytokine‐producing cell was not affected by fasting (Fig. 3B). However, fasting suppressed the levels of ILC2, effector CD4^+^ T cells and Th2 cytokines in the lungs and BALF (Fig. 4B and 4C). Although fasting did not affect the quality of Th2 cytokine‐producing cells, the amount of Th2 cytokines in BALF was suppressed by reducing the number of Th2 cytokine‐producing cells (ILC2 and effector CD4^+^ T cells) in the lungs.

During fasting starvation, cells activate AMP‐activated protein kinase (AMPK) in response to decreased glucose levels in blood and tissues. Activated AMPK acts as negative regulator of the mammalian target of rapamycin complex (mTORC) [32] and has a profound effect on cell proliferation, metabolism and survival. In particular, the inhibition of mTORC suppresses glucose transporter 1 (Glut1) expression [33] and switches energy metabolism from glycolysis to the TCA cycle and oxidative phosphorylation. Glut1 expression and glycolysis play important roles for differentiation and proliferation of effector T cells [34]. Thus, it can be assumed that fasting reduced the infiltration of eosinophils by suppressing the differentiation and proliferation of Th2‐producing cells (effector T cells). As another hypothesis, the gut microbiota is closely related to intestinal immunity and systemic immune function. Gut microbiota and metabolites regulate host immunity [35, 36]; however, the microbiota themselves are also affected by various factors such as diet, surrounding environment, and developmental stage [37, 38, 39]. As diet is an important factor that changes the composition of gut microbiota, a decrease in the gut energy caused by fasting may alter the diversity and abundance of the gut microbiota. Thus, fasting can affect the immune function of the host by altering the energy metabolism and gut microbiota of the host. Therefore, it is necessary to investigate whether these factors suppress the Th2 cytokine‐producing cells in the lungs.

**Fig. 5:**
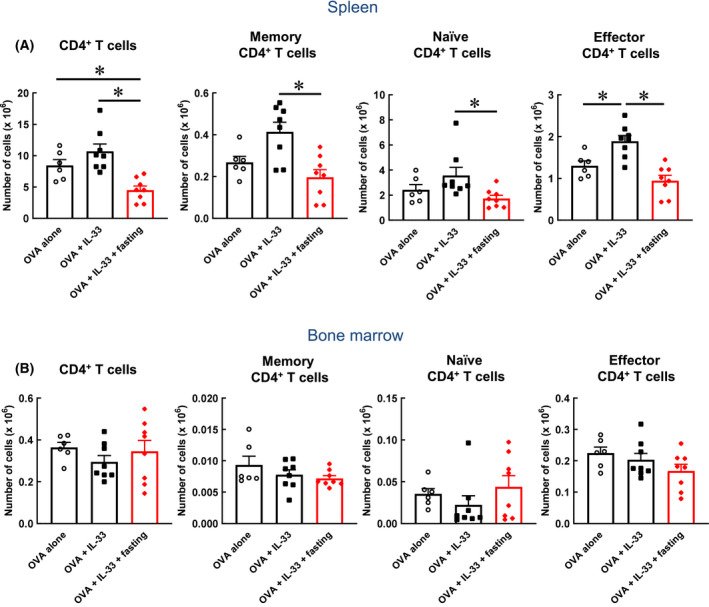
Effect of fasting on T cells in spleen and bone marrow. (A) The numbers of CD4^+^ T cell, memory CD4^+^ T cell, naïve CD4^+^ T cell and effector CD4^+^ T cell in the spleen were determined by flow cytometry. (B) The numbers of CD4^+^ T cell, memory CD4^+^ T cell, naïve CD4^+^ T cell and effector CD4^+^ T cell in the bone marrow were determined by flow cytometry. Each value represents the mean ± S.E.M. of 6–8 experiments. *P < 0.05 compared with each group. Statistical significance was determined by Scheffe’s test after performing an ANOVA test using StatView (SAS Institute, Inc., NC, USA). n.s.; not significant.

In this study, we demonstrated that T cells in the spleen were also suppressed because of fasting (Fig. 5A). This result suggests that the T cell suppression brought about by fasting is not a lung‐specific response. However, no such suppression of T cells was observed in the bone marrow (Fig. 5B). As the bone marrow functions as a reservoir of immune cells during fasting (Fig. 5B), some important immune cells might have survived because they were protected from fasting. Future studies must investigate the dynamics of the lung immune cells that decreased in concentration because of fasting.

In conclusion, fasting suppresses eosinophil infiltration by reducing the number of Th2 cytokine‐producing cells in the lung. These findings indicate that fasting can be used as a potential novel preventive treatment for eosinophilic asthma.

## Conflict of interest

The authors declare no conflict of interest.

## Author contributions

YS, TH, RY, FN, JI, TH, RO and KM had participated in the research design. YS, TH, RY, FN and JI had conducted the experiments. YS, TH, RY and FN had performed the data analysis. YS, TH and KM had drafted or contributed to the writing of the manuscript. TH and KM had supervised the experiments.

## Supporting information

**Fig. S1**. Gating strategy in flow cytometry analysis for immune cells in BALF. Gating strategy after the exclusion of doublets, dead fluorescent‐positive cells were shown. CD45^+^ Siglec‐F^+^ CD11c^‐^ cells (Eosinophil), CD45^+^ Siglec‐F^+^ CD11c^+^ cells (Alveolar macrophage), CD45^+^ CD11b^+^ Ly6G^‐^ cells (Monocyte), CD45^+^ CD11b^+^ Ly6G^+^ cells (Neutrophil), CD45^+^ CD4^+^ cell (CD4^+^ T cell), CD45^+^ CD8^+^ cells (CD8^+^ T cell) and CD45^+^ CD19^+^ cells (B cell) were analysed by flow cytometry.

**Fig. S2**. Gating strategy in flow cytometry analysis for the pulmonary infiltrates of ILC2 and effector CD4+ T cells. Gating strategy after the exclusion of doublets, dead fluorescent‐positive cells were shown. CD45^+^ ST2^+^ Lineage^‐^ Thy‐1.2^+^ Sca‐1^+^ cells (ILC2), CD45^+^ CD4^+^ cell (CD4^+^ T cell), CD45^+^ CD4^+^ CD44^+^ CD62L^‐^ cells (Effector CD4^+^ T cell), CD45^+^ CD4^+^ CD44^+^ CD62L^+^ cells (Memory CD4^+^ T cell) and CD45^+^ CD4^+^ CD44^‐^ CD62L^+^ cells (Naïve CD4^+^ T cell) were analysed by flow cytometry.

## Data Availability

The data that support the findings of this study are available from the corresponding author upon reasonable request and upon clearance from the Kumamoto University.
