# Supplementary figures and images for: Fasting impairs type 2 helper T cell infiltration in the lung of an eosinophilic asthma mouse model
Source: FEBS Open Bio. 2021 Aug 20;11(9):2619–30. doi: 10.1002/2211-5463.13268 (PMC8409288; doi:10.1002/2211-5463.13268)

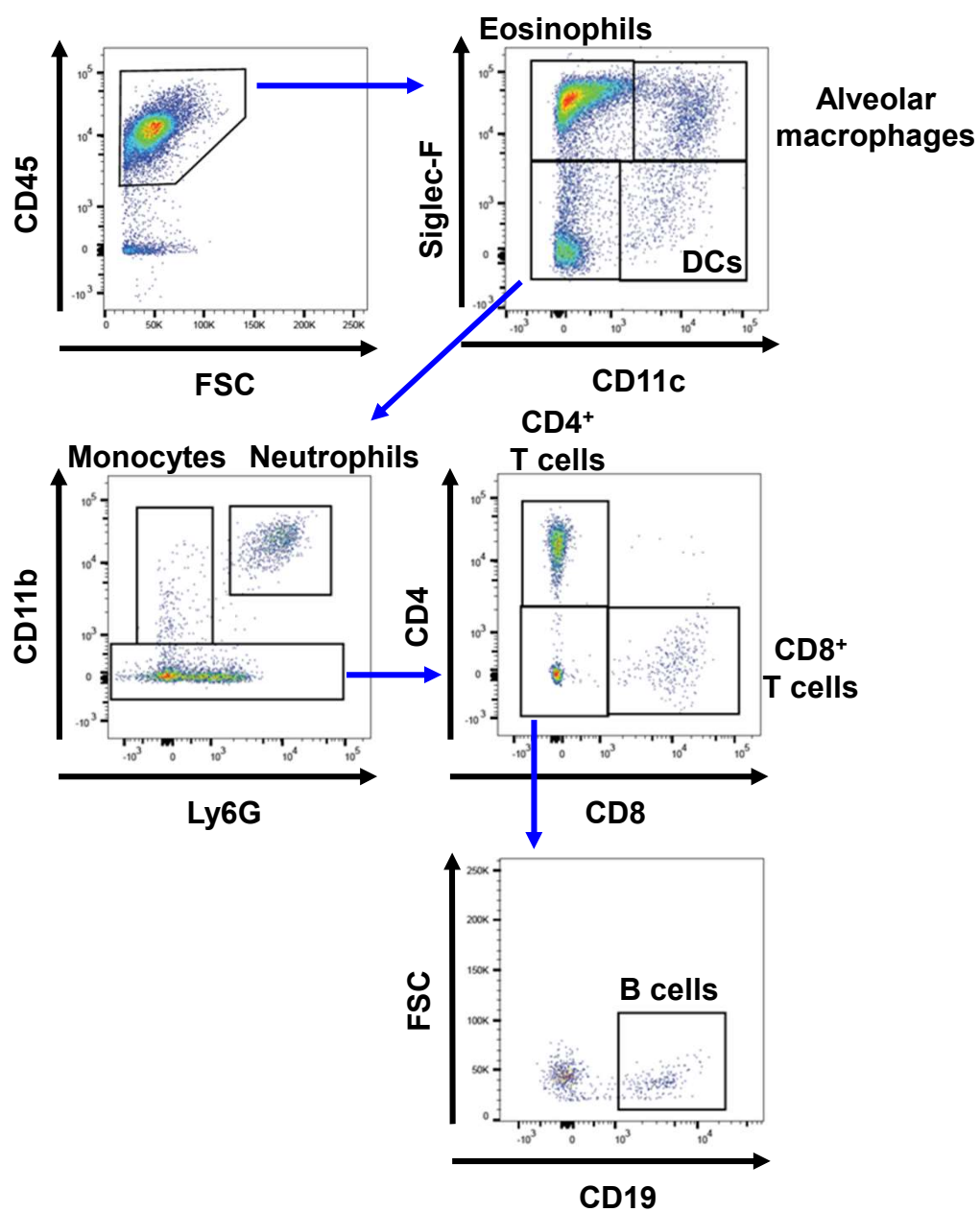

Supplementary Figure 1

Supplement: Supplementary file 1 — Fig. S1. Gating strategy in flow cytometry analysis for immune cells in BALF. Gating strategy after the exclusion of doublets, dead fluorescent‐positive cells were shown. CD45+ Siglec‐F+ CD11c‐ cells (Eosinophil), CD45+ Siglec‐F+ CD11c+ cells (Alveolar macrophage), CD45+ CD11b+ Ly6G‐ cells (Monocyte), CD45+ CD11b+ Ly6G+ cells (Neutrophil), CD45+ CD4+ cell (CD4+ T cell), CD45+ CD8+ cells (CD8+ T cell) and CD45+ CD19+ cells (B cell) were analysed by flow cytometry. [file FEB4-11-2619-s002.pdf]

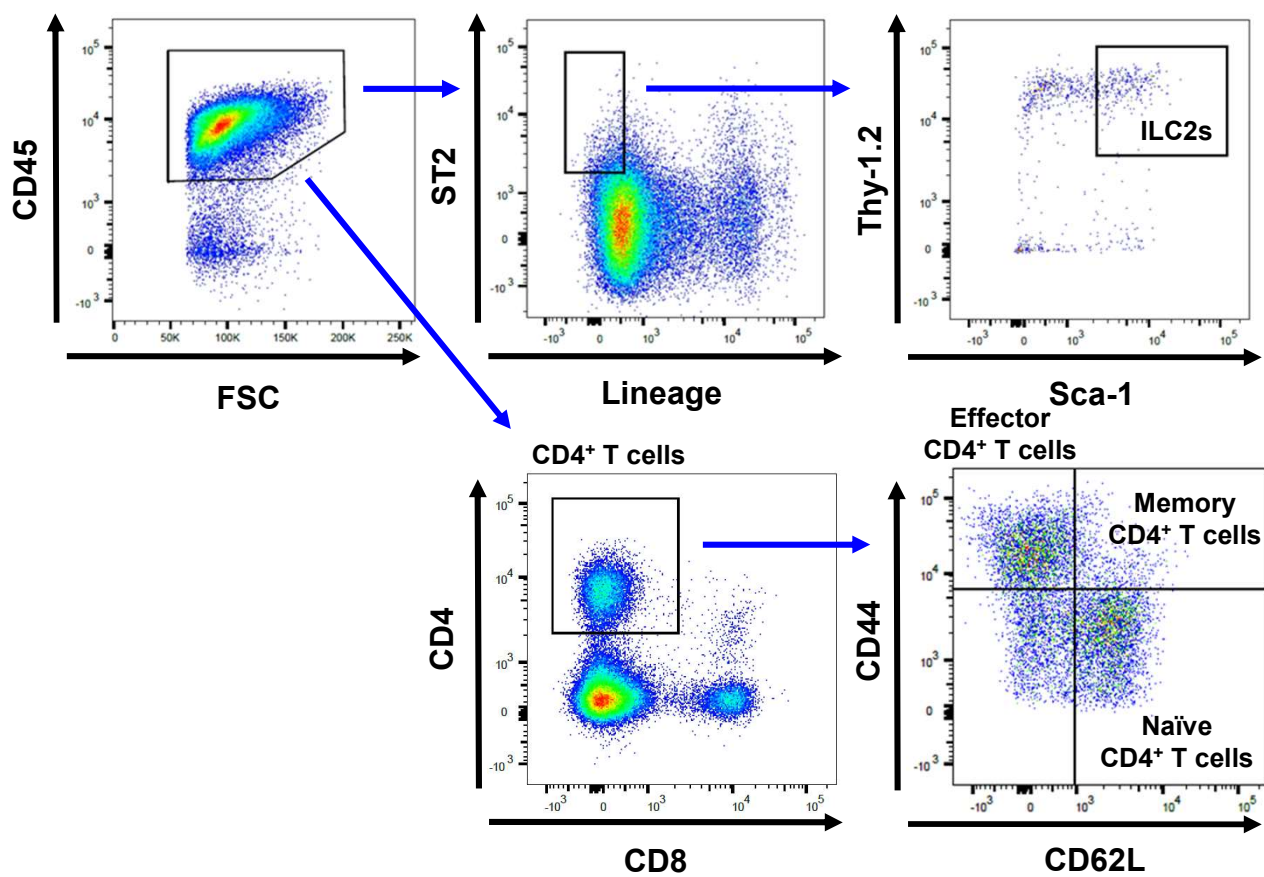

Supplementary Figure 2

Supplement: Supplementary file 2 — Fig. S2. Gating strategy in flow cytometry analysis for the pulmonary infiltrates of ILC2 and effector CD4+ T cells. Gating strategy after the exclusion of doublets, dead fluorescent‐positive cells were shown. CD45+ ST2+ Lineage‐ Thy‐1.2+ Sca‐1+ cells (ILC2), CD45+ CD4+ cell (CD4+ T cell), CD45+ CD4+ CD44+ CD62L‐ cells (Effector CD4+ T cell), CD45+ CD4+ CD44+ CD62L+ cells (Memory CD4+ T cell) and CD45+ CD4+ CD44‐ CD62L+ cells (Naïve CD4+ T cell) were analysed by flow cytometry. [file FEB4-11-2619-s001.pdf]
